# Reducing the Number of Individuals to Monitor Shoaling Fish Systems – Application of the Shannon Entropy to Construct a Biological Warning System Model

**DOI:** 10.3389/fphys.2018.00493

**Published:** 2018-05-08

**Authors:** Harkaitz Eguiraun, Oskar Casquero, Asgeir J. Sørensen, Iciar Martinez

**Affiliations:** ^1^Department of Graphic Design & Engineering Projects, Faculty of Engineering in Bilbao, University of the Basque Country UPV/EHU, Bilbao, Spain; ^2^Research Centre for Experimental Marine Biology and Biotechnology – Plentziako Itsas Estazioa, University of the Basque Country UPV/EHU, Plentzia, Spain; ^3^Department of Systems Engineering and Automatic Control, Faculty of Engineering in Bilbao, University of the Basque Country UPV/EHU, Bilbao, Spain; ^4^Centre for Autonomous Marine Operations and Systems, Department of Marine Technology, Norwegian University of Science and Technology, Trondheim, Norway; ^5^IKERBASQUE Basque Foundation for Science, Bilbao, Spain; ^6^Norwegian College of Fishery Science, Faculty of Biosciences, Fisheries and Economics, University of Tromsø, Tromsø, Norway

**Keywords:** fish monitoring, biological warning systems, fish welfare, the 3Rs, Shannon entropy, non-linear signal processing, non-invasive monitoring, intelligent aquaculture

## Abstract

The present study aims at identifying the lowest number of fish (European seabass) that could be used for monitoring and/or experimental purposes in small-scale fish facilities by quantifying the effect that the number of individuals has on the Shannon entropy (SE) of the trajectory followed by the shoal’s centroid. Two different experiments were performed: (i) one starting with 50 fish and decreasing to 25, 13, and 1 fish, and (ii) a second experiment starting with one fish, adding one new fish per day during 5 days, ending up with five fish in the tank. The fish were recorded for 1h daily, during which time a stochastic event (a hit in the tank) was introduced. The SE values were calculated from the images corresponding to three arbitrary basal (shoaling) periods of 3.5 min prior to the event, and to the 3.5 min period immediately after the event (schooling response). Taking both experiments together, the coefficient of variation (CV) of the SE among measurements was largest for one fish systems (CV 37.12 and 17.94% for the daily average basal and response SE, respectively) and decreased concomitantly with the number of fish (CV 8.6–10% for the basal SE of 2 to 5 fish systems and 5.86, 2.69, and 2.31% for the basal SE of 13, 25, and 50 fish, respectively). The SE of the systems kept a power relationship with the number of fish (basal: *R*^2^= 0.93 and response: *R*^2^= 0.92). Thus, 5–13 individuals should be the lowest number for a compromise between acceptable variability (<10%) in the data and reduction in the number of fish. We believe this to be the first scientific work made to estimate the minimum number of individuals to be used in subsequent experimental (including behavioral) studies using shoaling fish species that reaches a compromise between the reduction in number demanded by animal welfare guidelines and a low variability in the fish system’s response.

## Introduction

Large scale production aquaculture platforms, such as offshore exploitations, alone or within offshore multipurpose structures, are considered to hold the key to solve some of the challenges that must be addressed in order to increase the total production and the efficiency of fish farming to provide food for the exponentially growing human population (Bostock et al., 2010; Anon, 2011; European Aquaculture Technology and Innovation Platform-Eatip, 2012; Kalogerakis et al., 2015; FAO, 2016). One requisite for the optimal functionality of such production platforms is the implementation of intelligent structures that should be able to identify, register and respond to changing external and internal environments. In answer to this need and to improve the farmer’s ability to monitor, control and document biological processes in fish farms by applying control-engineering principles, the concept of precision fish farming (PFF) has been introduced (Føre et al., 2018). Unfortunately, in contrast to the increasing amount of works devoted to the study of the physical and intelligent design of the farming structures, there are few published works devoted to the automatic monitoring of the real fish being farmed and to the integration of that information into the whole intelligent system (Eguiraun et al., 2015). Monitoring of the fish behavior is important for at least three main reasons: (i) to avoid escapes, (ii) for the early detection of abnormalities in their behavior that may be an indication of disease, parasites or the presence of contaminants that may compromise their health and wholesomeness, and (iii) to document the fish welfare during the production.

Fish cognition and behavior is a well-established research field (Vila Pouca and Brown, 2017 and references therein), and the characterisation of fish model systems’ behavior, using behavioral measurable changes, has found several practical applications, such as the detection of leaders in a group (Mwaffo et al., 2017), the identification of information flows within a school of fish (Crosato et al., 2018), the presence in the aquatic environment of contaminants including caffeine (Ladu et al., 2015), drugs (Liu et al., 2011), hypochlorite (Magalhães et al., 2007; Nimkerdphol and Nakagawa, 2008; Teles et al., 2015), methyl-mercury (Eguiraun et al., 2014, 2016), the Se:Hg molar ratio in their feeds (Eguiraun et al., 2018) and alterations in environmental parameters such as hypoxia, feeding regime (Polonschii et al., 2013), and high fish density (Papoutsoglou et al., 1998; Di Marco et al., 2008).

Consequently, and following Hellou’s (2011) recommen-dations to assess the environmental quality of water, Eguiraun et al. (2015) recommended the implementation of biological warning system (BWS) into aquaculture by using fish of the same species being cultivated as the system’s sensor. The working hypothesis was that undesirable agents capable of altering biochemical and/or physiological processes of the fish would also alter the Shannon entropy (SE) of the system in a quantifiable manner (as shown by Eguiraun et al., 2014), and that this alteration could be used as an indicator of a deviation from the desired working point established by the fish farmer. Once the farmer detects a deviation, a series of pre-established rules included in the obligatory Hazard Analysis and Critical Control Point plan of each facility must be followed.

Each cage may hold several hundred thousand fish in intensive farming, i.e., up to several million fish per farm (Føre et al., 2018), which complicates the monitoring of all fish for control purposes. Therefore, to implement the BWS in an effective manner, one alternative is to construct a small-scale facility with fish of the same characteristics and subject to the same conditions as those in the commercial farming cages. Such a small-scale monitoring set-up would resemble and impose similar demands to the set-up for experimental studies with fish. These demands include respecting the ethical principles (Russell and Burch, 1959) and legal framework (European Commission, 2010) concerning the 3Rs. These three Rs (3Rs), necessary for a more ethical use of animals in testing, were initially mentioned by Russell and Burch (1959). They stand for Replacement: the adoption of methods which avoid or replace the use of animals in research (for example the use of mathematical models to study animal behavior instead of using live organisms); Reduction: the application of methods to obtain adequate information from fewer animals, or to obtain more information from the same number of animals and Refinement: the use of methods to eliminate or minimize potential pain, suffering or distress, and enhance animal welfare for the animals used. The present work can only contribute to the Reduction in the number of individuals for procedures that demand the use of live fish. To identify this lowest possible number is in itself a challenge, since there is usually no explanation regarding the criteria used to select the number of fish in physiological and toxicological experiments. The studies published on the effect of perturbations on fish systems, as well as behavioral studies, use different numbers of fish: some use only one fish (Magalhães et al., 2007; Brodin et al., 2013), while others use three fish (Teles et al., 2015), five fish (Crosato et al., 2018), fewer than 15 fish (Krause, 1993; Huth and Wissel, 1994; Krause and Tegeder, 1994; Ladu et al., 2015), 18–40 fish (Sadoul et al., 2014), 19–26 fish (Eguiraun et al., 2016), 30–300 fish (Tunstrøm et al., 2013), and 81 fish (Eguiraun et al., 2014). However, we have not been able to find any publication providing any scientific explanation about the reasons that led the authors to use those particular numbers of individuals.

In order to select the number of fish to test, the shoaling nature of the species must be taken into consideration. Studies on the collective behavior of different species have indicated that many observed features of social interactions can be predicted assuming that the individuals follow behavioral rules that maximize their entropy (Mann and Garnett, 2015) and that the collective behavior is determined by the number of topologically interacting neighbors, as proposed by Ballerini et al. (2008). These authors reconstructed 3D positions of airborne birds in flocks of thousands of individuals and showed that their interactions were based on their topological, and not metrical, distance, i.e., each bird interacted on average with a fixed number of neighbors (6–7), and not with all the neighbors within a fixed metric distance. Examples of interactions are orientation toward other fish, collective swarming, schooling, or flocking behaviors. Thus, in flocking starlings, each individual topologically interacts with 6–8 neighbors and the interaction with about 10 neighbors speeds up the rate of convergence (both speed and time to initiate the flocking behavior) irrespective of the total size of the swarm (Shang and Bouffanais, 2014). Studies on the social behavior of fish (Hemelrijk, 2002) indicate that fish schooling behavior emerge from the interaction of at least four neighbors (Huth and Wissel, 1994) and Crosato et al. (2018) used five fish to examine their interactions during the performance of U-turns in a circular tank of water. Consequently, considering the shoaling nature of the European seabass (*Dicentrarchus labrax*), our hypothesis was that there would be critical differences between the SE of, on one hand, the basal (shoaling) behavior of the systems of only one fish and those of more than one fish, and, on the other, the SE of the response to the event (schooling) of systems with fewer than five fish and systems with five or more fish (Huth and Wissel, 1994).

Accordingly, and given that previous studies (Kadota et al., 2011; Liu et al., 2011; Spasic et al., 2011; Quach et al., 2013; Bae and Park, 2014; Eguiraun et al., 2014, 2016, 2018; Forlim and Pinto, 2014) have identified the SE of the system as a variable with the potential to serve for fish health and welfare monitoring, the present work was designed to understand how the variation in the fish number affects the system dynamics in order to answer the following research questions: (i) Does the SE of a fish system vary according to the number of fish? (ii) if it does vary, how is this relationship? and, finally (iii) is it possible to identify the lowest number of individuals which could be used in monitoring and/or experimental settings? To answer these questions two different experiments were performed: (i) one experiment starting with 50 fish and decreasing the number to 25, 13, and finally one fish, and (ii) a second experiment, studying the system with initially one fish, then adding one new fish per day during 5 days, and ending with five fish in the tank.

Based on the experimental results, the main scientific contribution of the present work is to provide a key piece of information to set up a BWS, namely the minimum number of fish necessary to be monitored. The last part of this study presents a theoretical BWS model that integrates all the empirical knowledge obtained in order to provide results, in a non-invasive manner, about the health status of monitored or experimental fish.

## Materials and Methods

### Ethics Statement

The experimental protocols and procedures conducted in the present experiment had been approved by The Ethical Committee of the University of the Basque Country UPV/EHU for Animal Welfare No. CEBA/285/2013MG.

### Animals and Acclimation Conditions

European sea bass (*Dicentrarchus labrax*) generously provided by Grupo Tinamenor (Cantabria, Spain) had been acclimated in the Research Centre for Experimental Marine Biology and Biotechnology – Plentzia Marine Station of University of the Basque Country UPV/EHU for 3 months in two flow-through 1,800 L epoxy-coated fiberglass tanks containing aerated, naturally sand filtered seawater pumped from the Cantabric Sea in the North of the Iberian Peninsula (43°24′49.5″N 2°57′06.5″W). During this period, the seawater conditions oscillated according to the natural environmental variation, and they were always within the values for optimal growth for the species. The fish were fed INICIO Plus feed from BioMar (56% crude protein, 18% crude fat) following the manufacturer specifications for fish size, biomass and water temperature.

The length and weight of the fish used in Experiment A are shown in **Table 1** and the approximate total biomass for Experiment B is shown in **Table 2**. Fish of this size are considered sexually immature (Pickett and Pawson, 1994; Fishbase.org, 2015).

**Table 1:** Experiment A.

*n* = 50 fish	Tank 1		Tank 2	
	Size [mm]	Weight [g]	Size [mm]	Weight [g]
Avg	159.5	36.02	158.1	35.28
Max	200.0	60.00	197.0	64.00
Min	135.0	18.00	130.0	17.00
Median	154.5	33.50	156.0	33.00
Total biomass		1,801		1,764

*Biomass at the beginning of the experiment. Tanks 1 and 2 were filled with 50 fish each. Data on individual fish are shown in Supplementary Data Sheet S1*.

**Table 2:** Experiment B.

Day number	Tank 1		Tank 2	
	Fish name	Total biomass [g]	Fish names	Total biomass [g]
1	*a*	77	*b*	78
2	*c*	51	*a,b*	155
3	*d*	53	*a,b,c*	206
4	*e*	58	*a,b,c,d*	259
5	*f*	53	*a,b,c,d,e*	312

*Daily biomass in Tank 1. The biomass in Tank 2 is an approximation estimated by adding the weights of the fish coming from Tank 1, but the individual fish were not taken out of the Tank 2 and weighted every day*.

### Experimental Conditions

The salinity was measured using a multiparametric meter HANNA HI98192 and the O_2_ saturation with the JBL O_2_ kit. Water temperature, pH, and ammonium were monitored daily in both tanks using a thermometer (±0.5°C), a CRISOM pH-meter Basic 20+ and Sera NH_4_-NH_3_ ammonium kit, respectively. The values are shown in **Table 3**. Water flow (fixed at 0.54 m^3^/h) and additional air supply diffused by stone were kept constant and were interrupted, in order to avoid artifacts in the images, only during the time necessary to record the fish. The experiments were performed in the period November-December during which only small variations were detected in the seawater temperature and pH following the usual seasonal changes.

**Table 3:** Water/environmental conditions.

	Min	Max
Temperature [°C]	16.9	18.5
pH	7.76	7.93
Ammonium	0.0	0.0
Water flow [m^3^/h]	0.54	0.54
Salinity [g/l]	33	33
O_2_ Saturation	>80%	>80%

*Minimum and maximum values in relevant seawater parameters during the experimental period (November–December). Daily values are listed in Supplementary Data Sheet S2*.

Two identical fiberglass tanks were used (100 cm × 100 cm × 90 cm) under direct white artificial light (2 × 58 W and 5,200 lm), avoiding the formation of shadows into the tanks and using the same light conditions in both. The tanks, equipped with a flow through system, were filled up to 81 cm from the upper border with 810 L of naturally sand filtered seawater. One camera was placed in each tank and exactly in the same position in both tanks, obtaining in both situations the same visual angle. The photoperiod was fixed at 12h/12h dark/light.

### Experimental Set-Up

Experiment A was performed reducing the number of fish to imitate the usual procedure in many physiological and toxicological experiments. The fish are exposed to a given condition or contaminant and every x-days a certain number of fish (usually between 10 and 20, depending on the type of analyses to be performed, their cost and the expected variability of the parameter measured) are removed and sacrificed to perform biochemical and histological analyses, while the rest remains in the tank. After a new period of x-days the same number of fish is removed and so on. This is usually done to examine the effect of the contaminant, or the treatment, along time. In addition, we were interested in having more replicates of the measurements in tanks with only one fish, because, if it was a reliable system, that would be the most convenient from the point of view of reducing costs and animal suffering, and because many protocols use only one-fish to perform diverse studies, as mentioned in the introduction. Therefore, Experiment B was designed with two purposes: firstly, to obtain more replicates from one-fish system, but using different individual fish, and, secondly, to study the behavior of the system for 1–5 individuals, since Experiment A did not cover than range. In both, A and B Experiments, however, the individuals came from a larger group of fish and had been acclimated for at least 23 h to the identical settings as those used for this study.

### Experiment A – Systems With 1 to 50 Fish

**FIGURE 1:**
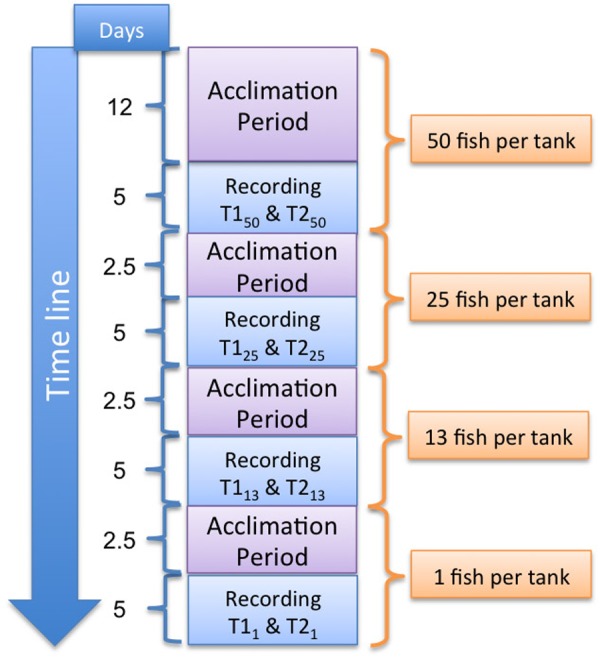
Description of Experiment A. The fish number is halved in each step except the last one, when it was reduced from 5 to 1 individual. T1 and T2 indicate tank 1 and 2, respectively, and the sub-index the number of fish. The number of days the activities lasted is shown under “Days” on the left.

Each of the two replicate groups consisted of 50 fish with a biomass as similar to each other as possible (**Table 1**). The fish were acclimated for 12 days to the new conditions, and they were monitored and recorded during the next 5 days following the procedure described below. After that, both groups were reduced to 25 fish, trying to maintain a similar biomass in both groups. The remaining 25 fish per group were acclimated for another 2.5 days and subsequently monitored and recorded for 5 days. Past those 5 days both groups were reduced to 13 fish per group, acclimated for 2.5 days and recorded for 5 days. Finally, the groups were reduced to only one fish. Again, after 2.5 days of acclimation, they were recorded for the final 5 days of the experiment (**Figure 1**).

### Experiment B – Systems With 1–5 Fish

**FIGURE 2:**
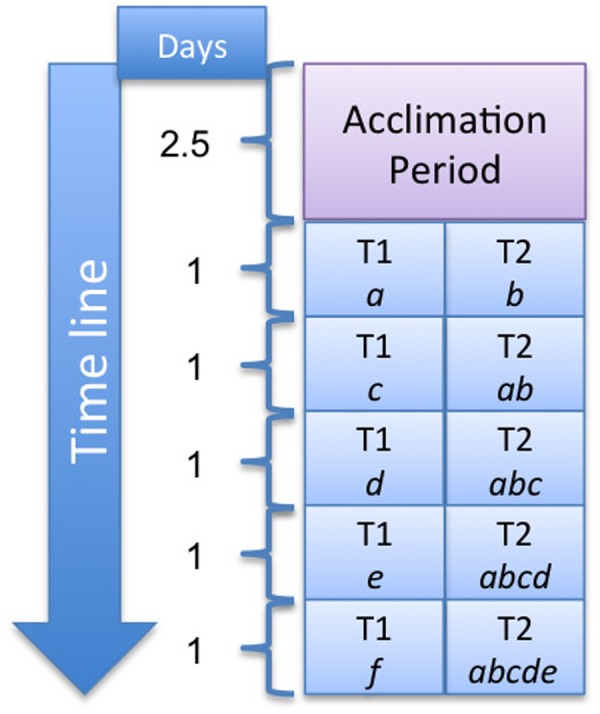
Description of Experiment B. The fish number is kept constant, only one fish, in tank 1. Each day the fish that had been for 1 day in tank 1 was transferred to tank 2. Thus, the number of fish in tank 2 increased by one individual every day. T1 and T2 indicate tank 1 and 2, respectively. The number of days the activity lasted is shown under “Days” on the left; each letter within the tanks, *a, b, c, d, e*, and *f* refer to an individual fish.

The experimental schedule is shown in **Figure 2**. In this particular case and during the 5 days the experiment lasted, tank 1 had only one fish and every day the fish that had been 1 day in tank 1 was transferred to tank 2, and a new fish was placed in tank 1. The new fish introduced every day in the experimental tank was taken from the acclimation tank not used for the experiments. All fish had an acclimation period of 23 h to the new experimental conditions. For a better understanding of the procedure, each fish has been named with a letter from *a* to *f* in **Figure 2** and **Table 2**. The approximate biomass is summarized in **Table 2**.

### Data Acquisition

Data acquisition was done by video camera as described in Eguiraun et al. (2014). In short, recording was performed using a GoProHero3 camera with underwater housing inside each tank. Raw data were recorded in 1080p high definition format, 24 frames per second (fps) and 16:9 video size and it was stored in SanDisk 32Gb UltraMicroSDHCTM (Class 10) secure cards.

**FIGURE 3:**
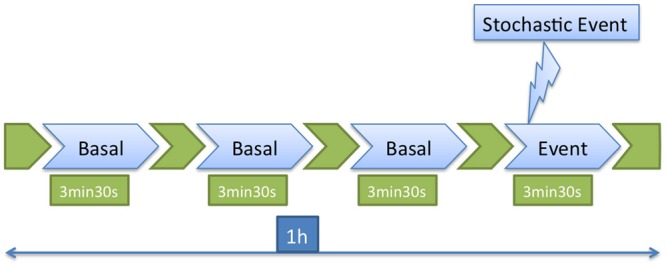
Recording procedure. Three basal and one event response measurements were processed from the total recorded period of 1 h.

As already mentioned, the water flow and air intake were halted during the recording period to avoid bubbles and disturbances in the images. Recording was set to 1 h per day and approximately in the middle of that period a stochastic event (a disturbance) consisting of a hit in the tank was introduced. The disturbance is a stochastic event, because it is meant to occur in a random manner, i.e., the fish must not be able to predict when it will take place The images to be processed consisted of three measures of the basal state, of 3.5 min each, and the 3.5 min after the disturbance, as described in Eguiraun et al. (2014) and in **Figure 3**.

### Image Post-processing

It was performed as described by Eguiraun et al. (2014). Once the four video clips (three arbitrary clips of the basal state and one clip containing the system’s response to the event) per tank and per day were located in the 1 h recording, they were transformed into a 640 pixel × 480 pixel format image sequences per video clip at 24 fps using the iMovie commercial software and MPEG Streamclip free software. Subsequent image and feature extraction were carried out with MATLAB R2014a (MathWorks Inc.) running on a MacBookPro 2,6 GHz Intel Core i7 laptop with a SSD storage disk and 16 Gb of RAM. The procedure used for image and feature extraction is detailed in Supplementary Data Sheet S3.

### Trajectory Estimation

**FIGURE 4:**
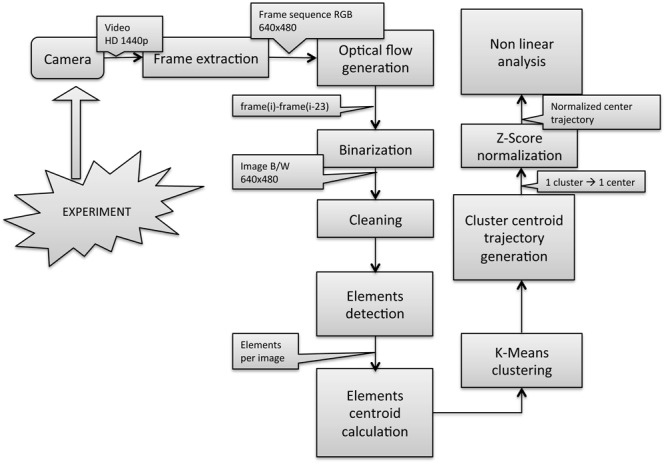
Data acquisition and processing workflow. Taken from Eguiraun et al. (2016).

The methodology used from image acquisition to fish group centroid trajectory estimation is depicted in **Figure 4** and was based on that described in Eguiraun et al. (2014) with the modifications detailed in Supplementary Data Sheet S4 and already used in Eguiraun et al. (2016). It was performed using MATLAB R2014a (MathWorks Inc.). Firstly, the trajectory of the cluster’s centroid was built computing the elements center’s in every single frame, which led to a very noisy signal unsuitable for the subsequent non-linear signal analysis. Thus, the noise of the signal was reduced calculating the cluster’s centroid applying the K-means algorithm to the number of elements in each frame using the centers of the elements in the first frame as input coordinates. Secondly, the trajectories in X and Y were analyzed in the same format they were obtained although they have different scale dimensions. X trajectories have dimension from 0 to 640 and Y trajectories have dimension from 0 to 480 due to the 640 × 480 pixel image size. The results indicated that analyzing those raw trajectories leads to satisfactory results and differences were not found between the results obtained analyzing the raw and the normalized trajectories. However, and with the purpose of building a more robust algorithm for future applications, the X and Y trajectories presented in the current work were normalized using Z-score technique. Supplementary Data sheet S5 contains the data for each of the 200 calculated trajectories.

### Non-linear Trajectory Analysis

Shannon entropy has been used because our previous work (Eguiraun et al., 2014) showed it to be the most sensitive among five algorithms tested, namely: Shannon and permutation entropies, and Katz, Higuchi, and Katz-Castiglioni’s fractal dimensions, to serve as a tool for the non-invasive quantification of fish responses and has subsequently been successfully applied to the study of the effect of certain chemicals (methylmercury and sodium selenite) on the complexity of the seabass centroid trajectories (Eguiraun et al., 2014, 2016, 2018). The Shannon entropy was initially described as an expression of the amount of missing information within a message, since the concept of entropy, within this particular context, was developed by Shannon in his works on a mathematical theory of communication (Shannon, 1948, 1951). Thus, the SE is a measure of the predictability of the value of a variable. The variable in our case is a time series consisting of samples constructed as successive positions of the fishes’ cluster’s centroid (x_i_,y_i_) in the frame of the image (640 pixels × 480 pixels; i.e., 0 < x_i_ < 640 and 0 < y_i_ < 480). If the predictability of the value of variables (*x* and *y*) is high, then the SE is low (i.e., if, knowing the values of *x*_i-1_ and *y*_i-1_, then it is easy to predict *x*_i_ and *y*_i_, respectively). On the other hand, the higher the difficulty to predict *x*_i_ and *y*_i_, the higher the SE. Thus, the highest SE will correspond to a system whose centroid may jump from any one position to any other one from frame to frame (i.e., all the pixels will have the same probability (1/640 will be the probability for every *x*_i_ and 1/480 for every *y*_i_). The lowest SE will correspond to a system whose centroid moves in a completely predictable manner: the centroid will occupy some few positions with a very high probability and the probability of occupying any other position will be practically zero. A real-life system will be somewhere between these two extremes.

We are aware of the fact that SE is not the optimal algorithm to explain sophisticated mental or behavioral processes, but we wish to stress that it is not our aim to study complex behavioral characteristics, as may be the orientation of the individuals, their interactions, how the shoal is formed, the presence of leaders, how the information flows among the individuals initiation and characteristics of collective behavior, etc. That kind of complex studies requires a completely different technical set up regarding image acquisition, data extraction, and analysis (see all the above mentioned papers on collective behavior and Gauvrit et al. (2017) for a recently published method of analysis for complex human behavior). We use the SE because we aim at implementing a system as simple and robust as possible and with the sole purpose of characterizing the trajectory signals of different experimental cases to perform comparisons among them. This very same simplicity, already described in our previous work (Eguiraun et al., 2014) and particularly regarding the 2D analysis of a 3D event together with the image segmentation method, makes our approach not suitable for complex behavioral studies, but adequate for routine monitoring of normal/not normal behaviors of the fish system, not of individual fish.

As already mentioned, the SE was first described by Shannon (1948, 1951) and it is calculated by the equation:

$$\begin{array}{c} H(X)\;=\;-\sum_{x_{i}\in \Theta }p(x_{i})\log p(x_{i})\;=\;-E[\log p(x_{i})] \end{array}$$

Where *X* represents a random variable with a set of values Θ and probability mass function p(x_i_) = P_r_{X = x_i_}, x_i_ ∈Θ, and *E* represents the expectation operator. Note that p log p = 0 if *p* = 0. The implementation in MATLAB R2014a (MathWorks Inc.) of the SE function is described in Supplementary Data Sheet S6.

### Statistical Parameters

The coefficients of variation (CV), defined as the ratio of the standard deviation to the mean, were calculated in Microsoft Office Excel 2007 and the curve fittings were performed using the Curve Fitting Toolbox 3.4.1 that is included in MATLAB R2014a (MathWorks Inc.).

## Results

**FIGURE 5:**
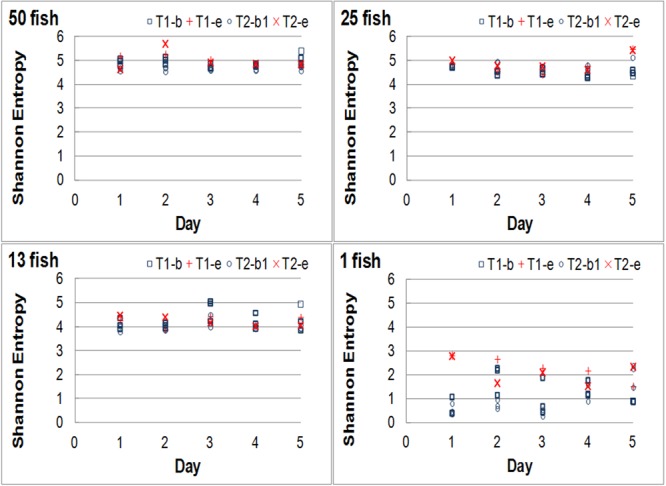
Daily evolution of the Shannon entropy (SE) for Experiment A showing the SE values obtained in tanks 1 and 2 for the basal state (T1-b and T2-b) and in response to the stochastic event (T1-e and T2-e). The number of individuals is indicated on the Top-Left of each plot.

**Table 4** and **Figure 5** show the daily evolution in both tanks of the SE corresponding to the basal trajectories (T1-b and T2-b in **Figure 5**) and to the trajectories followed in response to the stochastic event (T1-e and T2-e in **Figure 5**) of Experiment A. The responses obtained in both tanks were very similar and the SE of the system kept a power relationship with the number of fish (**Table 5**). In addition, the SE of the basal and response trajectories in tanks with 13 or more fish had always values higher than 3.97, while the in one-fish systems they were lower than 2.79. The coefficient of variation (CV) of the basal SE values also kept a relationship with the number of fish, being largest in the 1 fish systems (60.8% vs. about 4–8% for 50–13 fish, see **Table 6**). The raw data are listed in Supplementary Data Sheet S7.

**Table 4:** Daily evolution of the Shannon entropy in Experiment A in tanks 1 (T1) and 2 (T2).

		Day 1		Day 2		Day 3		Day 4		Day 5	
# fish	Tank #	Basal	Event	Basal	Event	Basal	Event	Basal	Event	Basal	Event
50	T_1_	4.92 ± 0.14	5.16	4.98 ± 0.15	5.23	4.73 ± 0.11	5.00	4.79 ± 0.04	4.77	5.09 ± 0.28	4.82
	T_2_	4.62 ± 0.07	4.62	4.66 ± 0.09	5.68	4.60 ± 0.04	4.89	4.63 ± 0.08	4.84	4.68 ± 0.10	4.81
25	T_1_	4.71 ± 0.01	4.85	4.47 ± 0.10	4.53	4.50 ± 0.14	4.41	4.30 ± 0.05	4.75	4.46 ± 0.11	5.46
	T_2_	4.76 ± 0.04	4.98	4.78 ± 0.22	4.76	4.67 ± 0.11	4.73	4.67 ± 0.10	4.58	4.69 ± 0.36	5.41
13	T_1_	4.11 ± 0.23	4.27	4.05 ± 0.11	3.88	4.75 ± 0.45	4.43	4.20 ± 0.34	4.05	4.34 ± 0.55	4.40
	T_2_	3.97 ± 0.17	4.47	3.99 ± 0.20	4.40	4.21 ± 0.27	4.16	3.99 ± 0.08	4.02	3.97 ± 0.08	4.06
1	T_1_	0.59 ± 0.39	2.79	1.84 ± 0.63	2.63	0.97 ± 0.77	2.26	1.34 ± 0.34	2.15	0.87 ± 0.03	1.49
	T_2_	0.52 ± 0.23	2.79	0.73 ± 0.19	1.63	0.38 ± 0.13	2.07	1.40 ± 0.47	1.50	2.01 ± 0.49	2.31

*Shannon entropy (SE) of the basal (average of six different measurements; two tanks and three measurements per tank) and event responses are shown. The raw data are listed in Supplementary Data Sheet S7*.

**Table 5:** Curve fitting parameters and goodness of the fit of the Shannon entropy (SE) vs. fish number.

y = a.x^b^ + c		Basal		95% confidence bounds	Response		95% confidence bounds
**For 1–5 fish systems**
Coefficients	*a*	–143.6		–22700, 22400	–0.92		–11.77, -0.06
	*b*	–0.01		–1.48, 1.47	–1.89		–7.80, 4.01
	*c*	145.10		–22400, 22700	3.42		2.60, 4.23
Goodness of the fit	SSE		8.07			0.42
	R^2^		0.72			0.79
	Adjusted R^2^		0.70			0.73
	RMSE		0.55			0.24
**For 1–50 fish systems**	
Coefficients	*a*	–4.17		–4.70, -3.64	–5.92		–11.77, -0.06
	*b*	–0.49		–0.64, -0.34	–0.16		–0.38, 0.05
	*c*	5.40		4.87, 5.94	8.21		2.31, 14.10
Goodness of the fit	SSE		28.24			5.90
	R^2^		0.93			0.92
	Adjusted R^2^		0.92			0.91
	RMSE		0.44			0.35

*The SE values (y) of the basal state and of the response to the event were fitted as a function of the fish number (x). a, b, and c are the coefficients of the curve. The goodness of the fit was estimated by the sum of squares due to error (SSE), R-square, adjusted R-square, and root mean squared error (RMSE).*

**Table 6:** Coefficients of variation (CV) of the measured SE values for Experiments A and B.

			Number of fish							
		**SE**	**50**	**25**	**13**	**5**	**4**	**3**	**2**	**1**
Experiment A, all measurements		Basal	4,19	4,34	8,10					60.83
Experiment B, all measurements		Basal				9,90	10,03	8,63	9,85	43.66
Experiments A+B										
All measurements		Basal	4,19	4,34	8,10	9,90	10,03	8,63	9,85	55,15
AVG of three replicates		Basal	2,31	2,69	5,86					37,12
All measurements		Response	6,12	7,20	4,93					19,51

*The CV of “All measurements” were calculated including all the SE values obtained, i.e., three basal and one response for each day. The “AVG of 3 replicates” were calculated including only the average of the three basal replicates obtained each day. There were no replicates of the responses to the event*.

**FIGURE 6:**
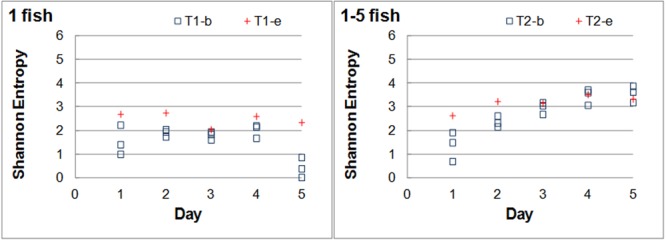
Daily evolution of the Shannon entropy in Experiment B. Tank 1 (Left plot) contained only one fish, but a different fish every day, during the 5 experimental days. The number of fish in Tank 2 (Right plot) increased by one individual daily. The number of fish is indicated on the Top-Left of the panels. The square markers correspond to the SE values for the basal states and the crosses to the SE in response to the event.

The results of Experiment B are shown in **Figure 6**. As in Experiment A, the SE of one-fish systems always kept similarly low values (lower than 2.2 for the basal and 2.7 for the response) and the SE of both the basal and response trajectories increased with increasing number of fish (**Figure 6**) following a power function (**Table 5**). Also as in Experiment A, the CV of the SE in the one-fish systems was much larger than in any of the other ones: 43.7% vs. about 10% for the 2–5 fish systems (**Table 6**). The raw data are listed in Supplementary Data Sheet S8.

**FIGURE 7:**
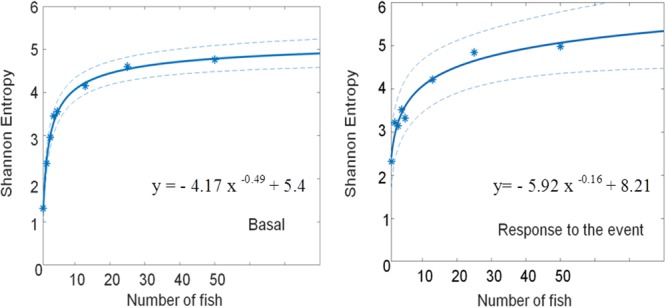
Curve fitting of the Shannon entropy as a function of the fish number. The basal state (Left) and the response to the event (Right) are shown together with the 95% confidence bounds. The parameters of the curve fitting are shown in **Table 5**.

Taken the results of both experiments together improved the goodness of the fit of the power relationship between the SE and number of fish (*R*^2^= 0.93 for the basal and *R*^2^= 0.92 for the response) and confirmed the higher variability in the SE of low-fish number systems, particularly those with only one-fish (**Tables 5, 6** and **Figure 7**).

### BWS Model

The purpose of measuring the SE of the basal and of the disturbed states was to obtain information on two relevant statuses (i.e., shoaling and schooling) in a healthy system in order to be integrated in a BWS. Since we found that both SE values kept a similar relationship with the number of fish, but they were not identical (**Figure 7**), we considered that the inclusion of both might strengthen a potential model that would ultimately permit their integration into a BWS monitoring tool, an example of which is described below. As already mentioned, our hypothesis, supported by previous works on the alteration of the SE in seabass systems contaminated with MeHg (Eguiraun et al., 2014, 2018), is that those SE values would be different in a healthy system than in an unhealthy one, and that this information may make possible to construct a model for a BWS.

**FIGURE 8:**
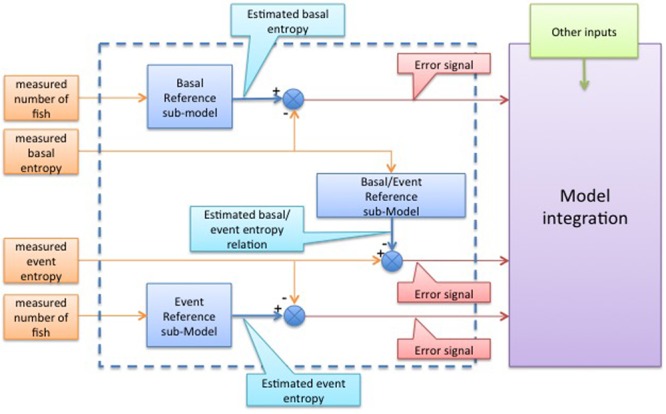
Schematic representation of the model defining inputs, generated outputs due to error signals, internal variables, and sub-model interactions. The output error signals should feed the subsequent phase of the model where all this information is integrated in the Model Integration box.

The first step in the construction of the model would be the collection of data corresponding to the healthy system under the same conditions in which the monitoring is going to be performed. These data include the size and number of the fish, and all the other environmental parameters. From these data, the SE of both the basal and disturbed states of both the control (healthy) system and of the system being monitored should be estimated. Using these four measurements, three sub-models would be constructed whose combination would provide the integrated or “overall” model, as shown in **Figure 8**. The three sub-models are: (i) Basal reference sub-model: built using the entropy generated by the fish system in its basal state; (ii) Event reference sub-model: built using the entropy of the fish system in response to a disturbance; (iii) Basal/Event relationship reference sub-model: built using the ratio between the “basal” and the “event” SE values.

The difference between the expected SE of the healthy system and the online signals measured by the monitoring tool detecting the actual SE of the system (for both the basal and disturbed statuses) will be called “error signal.” These error signals are the outputs of the proposed “overall” model and they should be integrated in knowledge models of higher order, i.e., as inputs to the “Model Integration” block in **Figure 8**. Error signals larger than the previously estimated normal variation in a healthy system should be interpreted as a deviation from the norm in the system (i.e., the introduction of a possibly undesirable agent) and the supervisor in charge of the monitoring operation should proceed to identify the cause of such deviation and follow the previously established corrective actions. Since it is desirable that the normal variation is a low as possible, one-fish systems (with CV of up to 60%) should be avoided.

## Discussion

The aim of the present work was to obtain an essential piece of information for BWS design purposes and for physiological research: to elucidate whether the number of fish affected the SE of the system in a known shoaling fish species (European seabass) and, if so, what type of relationship these two variables kept. As we have already mentioned, it must be noted that we did not aim at mapping behavioral characteristics such as time swimming or resting, aggressive behavior, the kind of shoaling and schooling itself or inter-individual interactions which would require a different methodological approach and algorithms more sophisticated than the SE to analyze the data (see for example the works by Tunstrøm et al., 2013; Teles et al., 2015; Gauvrit et al., 2017; Crosato et al., 2018).

The two experiments performed, A and B, are considered to contribute equally to the study of the system’s behavior with different number of fish, and whether the experiment was performed by decreasing or by increasing the number of fish in the tank should not have a bearing on the results because the fish had been acclimated for a long enough period of time prior to the recordings. The acclimation periods we have used (12 and 2.5 days and 23 h) are longer or similar to most of those reported in the literature, for example Stienessen and Parrish (2013) used only 1 day and Melvin et al. (2017) indicated that these kind of studies should be preceded by an acclimation period of at least several hours to evaluate normal baseline behaviors. For the freshwater species they used, mosquitofish (*Gambusia affinis*), this period was 8 h. Moreover, in both Melvin et al. (2017) and our present work, the fish had been acclimated to the laboratory tanks for 3 months prior to the initiation of the experiment, which was carried out in similar tanks and conditions to those to which they had been acclimated to.

Implementation of a BWS, or establishment of an experimental fish system, requires the characterization of the “normal” or “healthy” biological system, in order to be able to detect alterations provoked by the introduction of undesirable agents (such as predators, infectious or parasitic agents, contaminants or others) that would make the system become unbalanced, stressed or unhealthy. The healthy system will have a basal and a disturbed state, each with their corresponding SE values that will be “normal,” meaning that those will represent the shoaling basal state and the schooling reaction to a stochastic stimulus. The introduction of a detrimental agent (chronic stress, a toxicant, pathogen, parasite, etc.) should initiate the transformation of the “healthy” system into an “unhealthy” one and, consequently, induce alterations in the SE of both the basal and altered statuses. We initially thought that the SE in the response to a disturbance (i.e., the schooling reaction) might reflect better the health status of the system, so that if the fish had been affected for example, by a contaminant, its reflexes might have been altered and hence the initiation of the schooling should be different from the response of a healthy one (Eguiraun et al., 2014). On the other hand, it was also possible that the SE of the shoaling basal state itself might be different in healthy than in contaminated fish-systems and, in any case, it was likely that the information obtained from both measurements would be more robust than the information provided by any one of them. Therefore, we decided to include the analysis of both, the basal and disturbed states, in the present work and in the proposed model.

The number of fish is a characteristic intrinsic to the system and it should, a priori, not have a bearing on its health status. However, in the mid to long-term it may affect the health of the system if the number is too high or too low. Thus, in order to save costs, animal suffering and to respect the legal framework, it is desirable to select the lowest number that affects as little as possible the health status of the system, i.e., the lowest number of fish that provides results according to the variables being tested, such as contaminant concentration, and not to the composition of the system itself.

The one-fish systems had unusually low SE values, which may be explained by the shoaling nature of the species: when placed alone, we observed that fish moved very little, and this will translate in a low SE value due to the fact that it would be easier to predict the positions *x*_i_ and *y*_i_ (knowing *x*_i-1_ and *y*_i-1_) of a one-fish system that is hardly moving (we hypothesize that this is probably due to fear, as explained in the next paragraph), than the centroid of a cluster of fishes feeling safer within a shoal of increasing size that will probably display increased unpredictability of movements due to either free, random swimming, or to schooling to escape predators. The increased difficulty in predicting the trajectory of the shoal would result in concomitantly increasing SE values, as shown in Tank 2 of Experiment B and in Experiment A.

To explain the above hypothesis, we would like to introduce the selfish herd theory proposed by Hamilton (1971), according to which individuals in a herd will try to avoid the periphery where the risk of predation is greatest. This theory was empirically proven in a situation of stress for the minnow (*Phoxinus phoxinus*) (Krause, 1993) and for sticklebacks (*Gasterosteus aculeatus*) (Krause and Tegeder, 1994). Applying this theory, it is reasonable to assume: (i) that a fish which would naturally shoal, being alone in a clean tank where it cannot even exhibit full mimicry, will feel exposed and stressed, and will try to move as little as possible to avoid attracting undesired attention from potential predators, (ii) that the intensity of the response to this stress will vary according to the individual genotype, stock of origin and possibly life history of the individual, as shown by Herbert-Read et al. (2017), and (iii) that those factors will, in turn, contribute to the large CV of individual SE values of 1-fish systems, which must necessarily reflect the variability in the responses from each different individual.

Consequently, we would not recommend performing physiological or toxicological experiments, nor set up a BWS, with only one fish, given that the set up itself will likely influence the well being of the individual. Rather, it should be selected a system with the lowest number of fish that allows the individuals to feel safe, i.e., the lowest number that allow the fish feel that they are in a shoal and with possibilities to school and escape predators if necessary. For our fish and experimental conditions, somewhere between 5 and 13 fish would be acceptable. It is interesting to note that this number agrees with the 6–10 number of interacting neighbors to initiate convergence to swarming in birds (Shang and Bouffanais, 2014) and with the “at least four neighbors” necessary to achieve schooling behavior in fish (Huth and Wissel, 1994) previously mentioned.

The concepts and results shown here may apply not only to European seabass, but also to other similarly shoaling species. Although the behavior and response of the system will likely be species-specific, this approach might be applied with few modifications to monitoring species such as salmon, seabream, charr, cod, trout, and others of high relevance to the aquaculture industry. Furthermore, once the number of fish to be used in live systems has been scientifically selected, complex behavioral studies may be carried out using some of the more sophisticated analytical methods described by different authors and software (see references from the section “Introduction,” “Material and Methods,” and the free available software^1^). The main use of the present work would be to contribute to animal welfare and to scientifically justify the selection of the lowest possible number of individuals to be experimented upon when applying for the permit to perform experiments to the respective Animal Welfare Committees.

## Conclusions

We believe this to be the first scientific work designed to estimate the minimum number of individuals to be used in studies of shoaling fish species (albeit not of the shoaling itself) that reaches a compromise between the Reduction in number demanded by animal welfare guidelines and a low (or as low as possible) variability in the fish system’s response. This work also presents for the first time a potential model using the SE of the biological system, for the robust and practical implementation of a small-scale BW-monitoring system (to monitor the health and welfare of the fish) into an intelligent aquaculture platform.

Several conclusions can be drawn from the present study. One is that to set-up a monitoring BWS or an experiment using a shoaling species such as the European seabass, one should avoid using 1-fish systems. The second is that the minimum number of fish to monitor should be between five and 13 fish since that number is a good compromise between acceptable variability in the results and the concept of Reduction to satisfy the criteria for animal welfare in experimental settings. A third conclusion is that one should use both the basal SE and the SE in response to an event in the design of the practical model, since they give complementary information and both parameters are relevant. Finally, there is still a significant amount of work that needs to be done, as described in the next section, in order to further develop the BWS approach in practical aquaculture settings and, in particular, in Intelligent Aquaculture structures.

### Future Work

Further work within this line of research should include the validation of the present results using individuals of different size and species, as well as the development and validation of an early response model (such as the one presented above) of the system integrating all the relevant information needed to establish the “normal” response of the system. Once the monitoring system is defined, the next step will be its integration within the intelligent aquaculture structure. Additionally, it must be borne in mind that data on the system’s SE can be obtained by processing images, as we have done here, but infrared images, echo signals and labels carried by the fish are also methods with potential to provide such relevant information that have been tested and offer great promise (Føre et al., 2011, 2017). Last, but not least, the use of more complex methods for the acquisition of behavioral data and of algorithms for their analysis may provide further evidence as to the type of disturbance that may affect the system, when such disturbance takes place. The current procedure is only designed to identify a normal operating system from a deviated one, which may be enough for the farmer or the researcher to identify the presence of an agent causing an alteration into the system but, as mentioned above, more fine analyses might help to elucidate the type of alteration suffered and/or the type of external agent introduced that one should look for. The latter may be particularly interesting in the case of novel or unexpected contaminants.

It is a challenge to speculate on how a very large fish system, for example with several hundred thousand fish, may behave. This question, however, is very important if one wishes to optimize, in a rational manner, the building of large off-shore fish aquaculture structures. Whether it is the SE or some other better suited algorithm the one that may help us to understand the dynamics of such large systems and optimize them and the welfare of the fish, we cannot say at this time, but it is with no doubt a very interesting and challenging field of research that will contribute practical data to fish farmers. Future works in this field will require the contribution from experts with wide and very different fields of expertise.

## Author Contributions

HE conceived and designed the experiments, analyzed and interpreted the data, and wrote the manuscript. OC and AS interpreted the data and wrote the manuscript. IM contributed to the experimental design, analysis and interpretation of the data, and writing of the manuscript.

## Conflict of Interest Statement

The authors declare that the research was conducted in the absence of any commercial or financial relationships that could be construed as a potential conflict of interest.

## Footnotes

1 http://complexitycalculator.com
