# Supplementary material for: Reducing the Number of Individuals to Monitor Shoaling Fish Systems – Application of the Shannon Entropy to Construct a Biological Warning System Model
Source: Front Physiol. 2018 May 8;9:493. doi: 10.3389/fphys.2018.00493 (PMC5952214; doi:10.3389/fphys.2018.00493)
Supplement: Supplementary file 1 [file Data_Sheet_1.DOCX]

**S1. Experiment A fish** **biomass in the beginning of the experiment**. Tanks 1 and 2 were filled with 50 fish each.

|  | TANK 1 | | TANK 2 | |
| --- | --- | --- | --- | --- |
| Fish n# | **Length [mm]** | **Weight [g]** | **Length [mm]** | **Weight [g]** |
| 1 | 159 | 34 | 170 | 42 |
| 2 | 140 | 23 | 137 | 26 |
| 3 | 150 | 29 | 152 | 31 |
| 4 | 164 | 38 | 140 | 27 |
| 5 | 152 | 36 | 147 | 31 |
| 6 | 136 | 18 | 157 | 34 |
| 7 | 154 | 34 | 152 | 32 |
| 8 | 182 | 52 | 177 | 51 |
| 9 | 152 | 32 | 157 | 32 |
| 10 | 200 | 58 | 179 | 50 |
| 11 | 179 | 49 | 162 | 35 |
| 12 | 140 | 24 | 155 | 32 |
| 13 | 140 | 23 | 155 | 36 |
| 14 | 139 | 23 | 180 | 49 |
| 15 | 145 | 30 | 160 | 39 |
| 16 | 150 | 29 | 170 | 40 |
| 17 | 155 | 35 | 130 | 22 |
| 18 | 150 | 32 | 145 | 27 |
| 19 | 140 | 26 | 145 | 23 |
| 20 | 165 | 39 | 144 | 18 |
| 21 | 172 | 49 | 164 | 38 |
| 22 | 149 | 30 | 162 | 35 |
| 23 | 159 | 35 | 160 | 41 |
| 24 | 167 | 39 | 130 | 17 |
| 25 | 175 | 47 | 160 | 37 |
| 26 | 185 | 59 | 132 | 25 |
| 27 | 135 | 20 | 180 | 49 |
| 28 | 174 | 36 | 150 | 27 |
| 29 | 147 | 29 | 160 | 37 |
| 30 | 185 | 56 | 140 | 23 |
| 31 | 170 | 46 | 192 | 57 |
| 32 | 180 | 46 | 150 | 30 |
| 33 | 186 | 52 | 182 | 50 |
| 34 | 165 | 38 | 183 | 49 |
| 35 | 185 | 60 | 134 | 18 |
| 36 | 183 | 51 | 186 | 61 |
| 37 | 140 | 22 | 136 | 21 |
| 38 | 186 | 52 | 175 | 44 |
| 39 | 150 | 30 | 153 | 32 |
| 40 | 174 | 42 | 151 | 29 |
| 41 | 146 | 24 | 188 | 56 |
| 42 | 144 | 24 | 197 | 64 |
| 43 | 165 | 42 | 147 | 28 |
| 44 | 135 | 25 | 162 | 36 |
| 45 | 150 | 31 | 174 | 39 |
| 46 | 165 | 33 | 145 | 25 |
| 47 | 164 | 31 | 160 | 39 |
| 48 | 154 | 30 | 144 | 24 |
| 49 | 145 | 29 | 140 | 24 |
| 50 | 150 | 29 | 155 | 32 |
