# Supplementary material for: Reducing the Number of Individuals to Monitor Shoaling Fish Systems – Application of the Shannon Entropy to Construct a Biological Warning System Model
Source: Front Physiol. 2018 May 8;9:493. doi: 10.3389/fphys.2018.00493 (PMC5952214; doi:10.3389/fphys.2018.00493)
Supplement: Supplementary file 2 [file Data_Sheet_2.DOCX]

**S2.** **Water/environmental conditions**. Relevant seawater parameters during the experimental period (Nov-Dec 2014).

| Exp day | Phase | Tank | Fish n# | Water temp. [°C] | Ammonium | O_2_ | PH |
| --- | --- | --- | --- | --- | --- | --- | --- |
| 1 | Acclimation | T1 | 50 | 18.5 | 0 | 7 | 7.76 |
|  | Acclimation | T2 | 50 | 18.5 | 0 | 7 | 7.76 |
| 2 | Acclimation | T1 | 50 | 18.5 | 0 | 7 | 7.78 |
|  | Acclimation | T2 | 50 | 18.5 | 0 | 7 | 7.78 |
| 3 | Acclimation | T1 | 50 | 18.5 | 0 | 7 | 7.76 |
|  | Acclimation | T2 | 50 | 18.5 | 0 | 7 | 7.77 |
| 4 | Acclimation | T1 | 50 | 18.5 | 0 | 7 | 7.68 |
|  | Acclimation | T2 | 50 | 18.5 | 0 | 7 | 7.76 |
| 5 | Acclimation | T1 | 50 | 18.5 | 0 | 7 | 7.83 |
|  | Acclimation | T2 | 50 | 18.5 | 0 | 7 | 7.81 |
| 6 | Acclimation | T1 | 50 | 18.5 | 0 | 7.5 | 7.91 |
|  | Acclimation | T2 | 50 | 18.5 | 0 | 7.5 | 7.87 |
| 7 | Acclimation | T1 | 50 | 18.5 | 0 | 7.5 | 7.87 |
|  | Acclimation | T2 | 50 | 18.5 | 0 | 7.5 | 7.89 |
| 8 | Acclimation | T1 | 50 | 18.5 | 0 | 7.5 | 7.91 |
|  | Acclimation | T2 | 50 | 18.5 | 0 | 8 | 7.83 |
| 9 | Acclimation | T1 | 50 | 18.5 | 0 | 7 | 7.76 |
|  | Acclimation | T2 | 50 | 18.5 | 0 | 8 | 7.8 |
| 10 | Acclimation | T1 | 50 | 18.5 | 0 | 7 | 7.78 |
|  | Acclimation | T2 | 50 | 18.5 | 0 | 7 | 7.81 |
| 11 | Acclimation | T1 | 50 | 18.5 | 0 | 7 | 7.76 |
|  | Acclimation | T2 | 50 | 18.5 | 0 | 7 | 7.8 |
| 12 | Acclimation | T1 | 50 | 18.5 | 0 | 8 | 7.83 |
|  | Acclimation | T2 | 50 | 18.5 | 0 | 8 | 7.81 |
| 13 | A | T1 | 50 | 18.5 | 0 | 7 | 7.7 |
|  | A | T2 | 50 | 18.5 | 0 | 7 | 7.76 |
| 14 | A | T1 | 50 | 18.3 | 0 | 7 | 7.76 |
|  | A | T2 | 50 | 18.3 | 0 | 7 | 7.76 |
| 15 | A | T1 | 50 | 18.5 | 0 | 7 | 7.83 |
|  | A | T2 | 50 | 18.5 | 0 | 7 | 7.76 |
| 16 | A | T1 | 50 | 18.3 | 0 | 7 | 7.76 |
|  | A | T2 | 50 | 18.2 | 0 | 7 | 7.76 |
| 17 | A | T1 | 50 | 18.5 | 0 | 7 | 7.81 |
|  | A | T2 | 50 | 18.5 | 0 | 7 | 7.79 |
| 18 | Acclimation | T1 | 25 | 18.8 | 0 | 7 | 7.81 |
|  | Acclimation | T2 | 25 | 18.8 | 0 | 7 | 7.87 |
| 19 | Acclimation | T1 | 25 | 18.8 | 0 | 7 | 7.78 |
|  | Acclimation | T2 | 25 | 18.8 | 0 | 7 | 7.81 |
| 20 | A | T1 | 25 | 18.8 | 0 | 7 | 7.93 |
|  | A | T2 | 25 | 18.8 | 0 | 7 | 7.9 |
| 21 | A | T1 | 25 | 18.8 | 0 | 7 | 7.79 |
|  | A | T2 | 25 | 18.8 | 0 | 7 | 7.84 |
| 22 | A | T1 | 25 | 18.5 | 0 | 7 | 7.76 |
|  | A | T2 | 25 | 18.5 | 0 | 7 | 7.85 |
| 23 | A | T1 | 25 | 18 | 0 | 7 | 7.82 |
|  | A | T2 | 25 | 18 | 0 | 7 | 7.8 |
| 24 | A | T1 | 25 | 18 | 0 | 7 | 7.83 |
|  | A | T2 | 25 | 18 | 0 | 7 | 7.79 |
| 25 | Acclimation | T1 | 13 | 18 | 0 | 7 | 7.81 |
|  | Acclimation | T2 | 13 | 18 | 0 | 7 | 7.76 |
| 26 | Acclimation | T1 | 13 | 18 | 0 | 7 | 7.8 |
|  | Acclimation | T2 | 13 | 18 | 0 | 7 | 7.85 |
| 27 | A | T1 | 13 | 18.4 | 0 | 7 | 7.88 |
|  | A | T2 | 13 | 18 | 0 | 7 | 7.83 |
| 28 | A | T1 | 13 | 18 | 0 | 7 | 7.8 |
|  | A | T2 | 13 | 18 | 0 | 7 | 7.88 |
| 29 | A | T1 | 13 | 17.8 | 0 | 7 | 7.88 |
|  | A | T2 | 13 | 17.8 | 0 | 7 | 7.89 |
| 30 | A | T1 | 13 | 17.5 | 0 | 7 | 7.8 |
|  | A | T2 | 13 | 17.5 | 0 | 7 | 7.85 |
| 31 | A | T1 | 13 | 17 | 0 | 7 | 7.77 |
|  | A | T2 | 13 | 17 | 0 | 7 | 7.86 |
| 32 | Acclimation | T1 | 1 | 17 | 0 | 7 | 7.84 |
|  | Acclimation | T2 | 1 | 17 | 0 | 7 | 7.85 |
| 33 | Acclimation | T1 | 1 | 17 | 0 | 7 | 7.83 |
|  | Acclimation | T2 | 1 | 17 | 0 | 7 | 7.88 |
| 34 | A | T1 | 1 | 17 | 0 | 7 | 7.86 |
|  | A | T2 | 1 | 17 | 0 | 7 | 7.86 |
| 35 | A | T1 | 1 | 17 | 0 | 7 | 7.79 |
|  | A | T2 | 1 | 17 | 0 | 7 | 7.87 |
| 36 | A | T1 | 1 | 17 | 0 | 7 | 7.81 |
|  | A | T2 | 1 | 17 | 0 | 7 | 7.87 |
| 37 | A | T1 | 1 | 17 | 0 | 7 | 7.84 |
|  | A | T2 | 1 | 17 | 0 | 7 | 7.89 |
| 38 | A | T1 | 1 | 17 | 0 | 7 | 7.8 |
|  | A | T2 | 1 | 17 | 0 | 7 | 7.89 |
| 39 | Acclimation | T1 | 1a | 17 | 0 | 7 | 7.77 |
|  | Acclimation | T2 | 1b | 17 | 0 | 7 | 7.8 |
| 40 | Acclimation | T1 | 1a | 17 | 0 | 7 | 7.9 |
|  | Acclimation | T2 | 1b | 17 | 0 | 7 | 7.77 |
| 41 | B | T1 | 1b | 16.9 | 0 | 7 | 8 |
|  | B | T2 | 1a | 16.9 | 0 | 7 | 7.93 |
| 42 | B | T1 | 1ab | 16.9 | 0 | 7 | 7.9 |
|  | B | T2 | 1c | 16.9 | 0 | 7 | 7.93 |
| 43 | B | T1 | 1abc | 16.9 | 0 | 7 | 7.87 |
|  | B | T2 | 1d | 16.9 | 0 | 7 | 7.86 |
| 44 | B | T1 | 1abcd | 17 | 0 | 7 | 7.78 |
|  | B | T2 | 1e | 17 | 0 | 7 | 7.89 |
| 45 | B | T1 | 1abcde | 16.9 | 0 | 7 | 7.88 |
|  | B | T2 | 1f | 16.9 | 0 | 7 | 7.93 |
