# Supplementary material for: Reducing the Number of Individuals to Monitor Shoaling Fish Systems – Application of the Shannon Entropy to Construct a Biological Warning System Model
Source: Front Physiol. 2018 May 8;9:493. doi: 10.3389/fphys.2018.00493 (PMC5952214; doi:10.3389/fphys.2018.00493)
Supplement: Supplementary file 3 [file Data_Sheet_3.DOCX]

**S3.** **Matlab scripts indicating image and feature extraction.**

**%% Images from RGB to BW**

for i=10001:15300

I=imread(fullfile(sprintf('image_%d.png',i)));

J=imread(fullfile(sprintf('image_%d.png',i+24)));

%% Optical flux generation

H = J-I;

H_gray = rgb2gray(H);

H_gray=imadjust (H_gray,[0 1], [0 1], 0.7);

H_bw = im2bw(H_gray,0.07);

%% Erode and dilate

H_bw = bwmorph(H_bw,'clean');

H_bw = bwmorph(H_bw,'close');

H_bw = bwareaopen(H_bw,20);

%% Clean images

se1=strel('disk',3);

H2_bw = imclose(H_bw,se1);

%% Store

imwrite(H2_bw, fullfile(sprintf('finalimage_%d.png',i)),'png');

end

**%% Each frame characterization**

inicio = input('Enter the first number of the frame sequence:');

final = input('Enter the last number of the frame sequence:');

index = final-inicio;

Props(100,index) = struct( 'Area', [], 'Centroide', [], 'BoundingBox',[]);

Props(100,index).Area = []; Props(100,index).Centroide = []; Props(100,index).BoundingBox = [];

CoorX = zeros(100,index);

CoorY = zeros(100,index);

for ii=inicio:final

A = imread(fullfile(sprintf('imagefinal_%d.png',ii)));

iii=ii-(inicio-1);

[Iconex num]=bwlabeln(A,8);

data = regionprops(Iconex);

[x1,x2]=size(data);

elem(1,iii)=x1;

for j=1:x1

Props(j,iii).Area= data(j,1).Area;

Props(j,iii).Centroide=data(j,1).Centroid;

Props(j,iii).BoundingBox=data(j,1).BoundingBox;

CoorX(j,iii) = Props(j,iii).Centroide(1,1);

CoorY(j,iii) = Props(j,iii).Centroide(1,2);

end

end

if elem(1)==0

n = 1;

while elem(n)==0

n=n+1;

end

elem (1)=elem(n);

Props(1,1)=Props(1,n);

CoorX(1,1) = Props(1,1).Centroide(1,1);

CoorY(1,1) = Props(1,1).Centroide(1,2);

end
