# Supplementary material for: Reducing the Number of Individuals to Monitor Shoaling Fish Systems – Application of the Shannon Entropy to Construct a Biological Warning System Model
Source: Front Physiol. 2018 May 8;9:493. doi: 10.3389/fphys.2018.00493 (PMC5952214; doi:10.3389/fphys.2018.00493)
Supplement: Supplementary file 4 [file Data_Sheet_4.DOCX]

**S4.** **Matlab scripts indicating Trajectory estimation.**

**%% Image sequence clustering, trajectory estimation and entropy calculation**

inicio = input('Enter the first number of the frame sequence:');

final = input('Enter the last number of the frame sequence:');

filename = 'XXXX';

k = 1;

frames = index;

ClusterTray = zeros (frames,2*k);

for h=1:frames

finalelem = elem(1,h);

Coord=zeros (finalelem,2);

Coord(:,1) = CoorX(1:finalelem,1);

Coord(:,2) = CoorY(1:finalelem,1);

if finalelem == 1;

ClusterTray(h,1) = Coord(1,1);

ClusterTray(h,2) = Coord(1,2);

elseif finalelem == 0;

ClusterTray(h,1) = ClusterTray(h-1,1);

ClusterTray(h,2) = ClusterTray(h-1,2);

else

[IDX,Centr,sumd,D] = kmeans(Coord,k);

ClusterTray(h,1) = Centr(1,1);

ClusterTray(h,2) = Centr(1,2);

end

end

ClusterTrayX = zscore (ClusterTray(:,1));

ClusterTrayY = zscore (ClusterTray(:,2));

prec = -1;

HX = entropy(ClusterTrayX, prec);

HY = entropy(ClusterTrayY, prec);

save(filename,'ClusterTray','ClusterTrayX','ClusterTrayY','HX','HY')
