# Supplementary material for: Reducing the Number of Individuals to Monitor Shoaling Fish Systems – Application of the Shannon Entropy to Construct a Biological Warning System Model
Source: Front Physiol. 2018 May 8;9:493. doi: 10.3389/fphys.2018.00493 (PMC5952214; doi:10.3389/fphys.2018.00493)
Supplement: Supplementary file 6 [file Data_Sheet_6.DOCX]

**S6. Matlab function to calculate the Shannon entropy of each trajectory signal from the raw data listed in Supplementary 5.**

**% This function calculates the entropy of input vector.**

**% y - input column vector**

**% prec - precision of calculation; if prec = -1 then the precision will be maximal (default: depending on the length of vector)**

**% H - output entropy**

% Paths and variables

if((nargin < 2) || isempty(prec))

if(length(y) < 64)

prec = 32;

elseif(length(y) < 128)

prec = 64;

else

prec = 256;

end

end

% Calculate the probability

if(prec > 0)

p = hist(y, prec);

p = p./sum(p);

p = p(p > 0);

else

u_y = unique(y).';

p = sum(y(:,ones(1,length(u_y))) == u_y(ones(length(y),1),:))./length(y);

end

% Calculate the entropy

H = -sum(p.*log2(p));
