# Supplementary material for: Reducing the Number of Individuals to Monitor Shoaling Fish Systems – Application of the Shannon Entropy to Construct a Biological Warning System Model
Source: Front Physiol. 2018 May 8;9:493. doi: 10.3389/fphys.2018.00493 (PMC5952214; doi:10.3389/fphys.2018.00493)
Supplement: Supplementary file 7 [file Data_Sheet_7.DOCX]

**S7.** **Daily evolution of the Shannon entropy in Experiment A in tanks T1 and T2.** Shannon entropy of the basal and event responses are shown.

| Phase A | | | Day 1 | Day 2 | Day 3 | Day 4 | Day 5 |
| --- | --- | --- | --- | --- | --- | --- | --- |
| 50 fish | **T1** | **Basal1** | 4.9579 | 4.9922 | 4.6617 | 4.7448 | 4.8105 |
|  |  | **Basal2** | 4.7666 | 4.8261 | 4.6807 | 4.8347 | 5.0868 |
|  |  | **Basal3** | 5.0434 | 5.1223 | 4.8546 | 4.7939 | 5.3804 |
|  |  | **Event1** | 5.1619 | 5.2336 | 5.0027 | 4.7719 | 4.825 |
|  | **T2** | **Basal1** | 4.677 | 4.5164 | 4.5909 | 4.6055 | 4.5503 |
|  |  | **Basal2** | 4.6524 | 4.686 | 4.5745 | 4.5624 | 4.7067 |
|  |  | **Basal3** | 4.5449 | 4.675 | 4.648 | 4.7256 | 4.7463 |
|  |  | **Event1** | 4.6186 | 5.6768 | 4.891 | 4.8404 | 4.8114 |
| 25 fish | **T1** | **Basal1** | 4.7243 | 4.3635 | 4.4236 | 4.2599 | 4.5763 |
|  |  | **Basal2** | 4.7007 | 4.5226 | 4.4163 | 4.3627 | 4.4577 |
|  |  | **Basal3** | 4.6972 | 4.5395 | 4.6643 | 4.2863 | 4.3546 |
|  |  | **Event1** | 4.85 | 4.5336 | 4.4098 | 4.7515 | 5.457 |
|  | **T2** | **Basal1** | 4.7771 | 4.5302 | 4.5406 | 4.5903 | 4.482 |
|  |  | **Basal2** | 4.7113 | 4.8928 | 4.7388 | 4.651 | 4.4855 |
|  |  | **Basal3** | 4.7886 | 4.9307 | 4.7262 | 4.7795 | 5.1064 |
|  |  | **Event1** | 4.9816 | 4.7627 | 4.7343 | 4.5846 | 5.4108 |
| 13 fish | **T1** | **Basal1** | 4.0651 | 4.0632 | 4.2345 | 3.912 | 3.8519 |
|  |  | **Basal2** | 3.9024 | 4.1638 | 4.9665 | 4.1263 | 4.2277 |
|  |  | **Basal3** | 4.3612 | 3.9362 | 5.0579 | 4.5757 | 4.9386 |
|  |  | **Event1** | 4.266 | 3.8806 | 4.4282 | 4.0546 | 4.3983 |
|  | **T2** | **Basal1** | 3.7781 | 3.8464 | 3.9799 | 3.9075 | 3.9212 |
|  |  | **Basal2** | 4.0262 | 3.893 | 4.1318 | 4.0582 | 3.9279 |
|  |  | **Basal3** | 4.0999 | 4.2196 | 4.5095 | 4.012 | 4.0682 |
|  |  | **Event1** | 4.4757 | 4.4021 | 4.1642 | 4.0177 | 4.0587 |
| 1 fish | **T1** | **Basal1** | 0.372 | 2.2432 | 0.4195 | 1.1708 | 0.8486 |
|  |  | **Basal2** | 0.3645 | 1.1193 | 0.6484 | 1.126 | 0.8586 |
|  |  | **Basal3** | 1.046 | 2.1668 | 1.8578 | 1.7398 | 0.9048 |
|  |  | **Event1** | 2.7932 | 2.6348 | 2.2591 | 2.1489 | 1.4923 |
|  | **T2** | **Basal1** | 0.7771 | 0.9343 | 0.5102 | 0.8702 | 2.3587 |
|  |  | **Basal2** | 0.4406 | 0.5637 | 0.2415 | 1.7718 | 2.2286 |
|  |  | **Basal3** | 0.3345 | 0.6814 | 0.3881 | 1.5647 | 1.4476 |
|  |  | **Event1** | 2.7588 | 1.6263 | 2.0664 | 1.5008 | 2.3106 |
