# Supplementary material for: Reducing the Number of Individuals to Monitor Shoaling Fish Systems – Application of the Shannon Entropy to Construct a Biological Warning System Model
Source: Front Physiol. 2018 May 8;9:493. doi: 10.3389/fphys.2018.00493 (PMC5952214; doi:10.3389/fphys.2018.00493)
Supplement: Supplementary file 8 [file Data_Sheet_8.DOCX]

**S8.** **Daily evolution of the Shannon entropy in Experiment B in tanks T1 and T2.** Shannon entropy of the basal and event responses are shown.

| Phase B | | | Day 1 | Day 2 | Day 3 | Day 4 | Day 5 |
| --- | --- | --- | --- | --- | --- | --- | --- |
| 1-5 fish | **T1** | **Basal1** | 0.6822 | 2.1497 | 2.6764 | 3.0638 | 3.1742 |
|  |  | **Basal2** | 1.4811 | 2.3174 | 3.0445 | 3.6018 | 3.6221 |
|  |  | **Basal3** | 1.9068 | 2.6086 | 3.1679 | 3.712 | 3.8685 |
|  |  | **Event1** | 2.6216 | 3.2212 | 3.1468 | 3.5152 | 3.3237 |
| 1 fish | **T2** | **Basal1** | 0.9907 | 1.7281 | 1.9329 | 1.6669 | 0.8534 |
|  |  | **Basal2** | 1.3949 | 1.9337 | 1.8254 | 2.136 | 0.0093 |
|  |  | **Basal3** | 2.2216 | 2.0328 | 1.5992 | 2.1928 | 0.3744 |
|  |  | **Event1** | 2.6858 | 2.7352 | 2.0313 | 2.5919 | 2.332 |
